# Evaluation of atrial volume and function with magnetic resonance imaging in hypoplastic left heart

**DOI:** 10.1186/1532-429X-15-S1-O41

**Published:** 2013-01-30

**Authors:** Chodchanok Vijarnsorn, Kimberley A Myers, David J Patton, Cinzia Crawley, Michelle Noga, Edythe B Tham

**Affiliations:** 1 Mazankowski Alberta Heart Institute, University of Alberta, Edmonton, AB, Canada; 2 Alberta Children's Hospital, Calgary, AB, Canada

## Background

Increased left atrial volume and reduced atrial emptying are predictors of adverse cardiac events in adults. The atrium in single ventricle hearts contributes to ventricular filling to a greater degree than in normal hearts. Standardized methods of determining atrial function in single ventricles are lacking. We aimed to assess the feasibility and reproducibility of quantifying atrial volumes and function in hypoplastic left heart (HLH) prior to the Glenn operation.

## Methods

**Figure 1:**
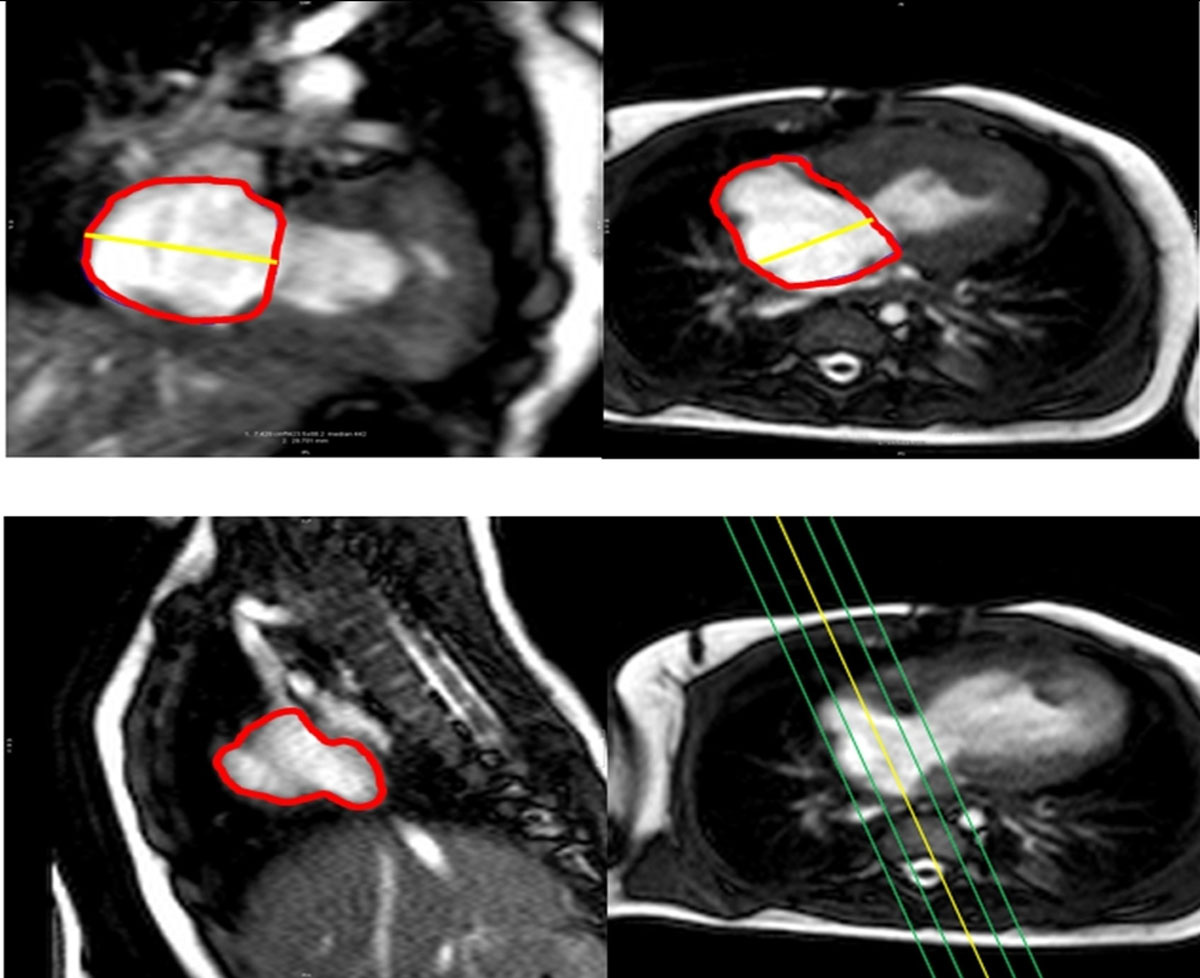
Measurement of right atrial volumes in hypoplastic left heart in 4CH, 2CH and SAO stack.

Fourteen patients with HLH prior to Glenn surgery (4.2 ± 1.2 months) underwent CMR cine imaging in 2 chamber (2CH) plane, 4 chamber (4CH) plane and 3D-atrial short-axial oblique (3D-SAO) stack (Figure 1). Right atrial volumes and atrial ejection fraction (aEF) were assessed by 3 methods: 1) biplane = 0.85 x area4CH x area2CH / (length4CH + length2CH)/2; 2) 2CH monoplane; and 3) 4CH monoplane area-length formula:=0.85xarea2/length in end-diastole (aEDV) and end-systole (aESV). These 3 methods were compared to 3D-SAO volume and aEF by paired t-test. Agreement was compared using mean differences (diff) and Spearman correlation. Interobserver reproducibility was analyzed by intra class correlation (ICC). Correlation of aEDV, aESV and aEF with RV mass was analyzed using Spearman correlation.

## Results

Monoplane 4CH and biplane methods showed high correlations for aEDV and aESV as well as small mean differences compared to 3D-SAO (Table 1). Despite a good correlation with 3D-SAO, the monoplane 2CH method underestimated aEDV and aESV. The aEF by all methods showed high correlations, but overestimated aEF when compared to 3D-SAO. Inter and intra observer variability demonstrated good agreement in both 3D SAO (aEDV: ICC 0.79, aESV:ICC 0.84, aEF 0.87; all p<0.001) and 4CH (EDV: ICC 0.73; p 0.001, ESV:ICC 0.68; p<0.001, aEF 0.50, all p<0.02). Increased RV mass index was related to increased atrial ESV index (R -0.67, p 0.03) and decreased aEF (R -0.76, p 0.01).

**Table 1:** Comparison of aEDV, aESV and aEF between 3D-SAO and monoplane 4CH, 2CH and biplane method (n=14)

	Mean	Mean differences (with 3D SAO)	p-value	Correlation (R)	p-value
aEDV(mL) - 3D SAO - 4CH - 2CH - biplane	15.77 ± 3.02 15.55 ± 4.06 13.68 ± 2.99 14.82 ± 3.02	- - 0.22 ± 3.01 - 2.08 ± 1.61 - 0.95 ± 1.67	- 0.78 <0.001 0.05	- 0.68 0.84 0.84	- 0.008 <0.001 <0.001

aESV (mL) - 3D SAO - 4CH - 2CH - biplane	8.73 ± 2.28 7.88 ± 2.61 7.37 ± 2.44 7.71 ± 2.37	- -0.85 ± 1.56 -1.35 ± 1.34 -1.01 ± 0.95	- 0.07 0.002 0.001	- 0.80 0.84 0.91	- 0.001 <0.001 <0.001

aEF - 3D SAO - 4CH - 2CH - biplane	0.45 ± 0.08 0.51 ± 0.11 0.47 ± 0.09 0.48 ± 0.08	- 0.06 ± 0.06 0.02 ± 0.04 0.03 ± 0.03	- 0.006 0.07 0.004	- 0.76 0.88 0.91	- 0.001 <0.001 <0.001

Values are expressed by mean ± SD Statistical significance at p value < 0.05

## Conclusions

CMR atrial volume assessment is feasible in HLH prior to Glenn operation. The monoplane 4CH and biplane methods show the best correlate of atrial volumes and function. Increased RV mass may be associated with reduced atrial function.

## Funding

None.

